# Numerical Investigation of Mixing Performance in Microfluidic Chip via Structural Micro-Rotors

**DOI:** 10.3390/mi16070806

**Published:** 2025-07-11

**Authors:** Yongliang Dong, Liqiu Wang, Xing Han

**Affiliations:** 1 Guangdong Provincial Key Laboratory of Sensing Technology and Biomedical Instrument, School of Biomedical Engineering, Shenzhen Campus of Sun Yat-Sen University, Sun Yat-Sen University, No. 66, Gongchang Road, Guangming District, Shenzhen 518107, China; 2 Department of Mechanical Engineering, The Hong Kong Polytechnic University, Hong Kong 999077, China

**Keywords:** microfluidics, active mixing, rotors, high-viscosity liquids

## Abstract

Microfluidics is a powerful tool with extensive applications, including chemical synthesis and biological detection. However, the limited channel size and high viscosity of samples/reagents make it difficult to fully mix liquids and improve the reaction efficiency inside microfluidic chips. Active mixing by rotors has been proven to be an effective method to promote mixing efficiency via a magnetic field. Here, we numerically investigated the mixing performance of rotors with different shapes (bar-shaped, Y-shaped, and cross-shaped). We systematically studied the influence of the arrangement of multiple cross-rotors and the rotation rate on mixing performance. The results are promising for instructing the design and manipulation of rotors for in-channel mixing.

## 1. Introduction

Microfluidics has been proven to be a powerful tool for various applications like biological detection, synthesis, and drug discovery [1,2,3,4,5,6,7,8]. Small volume and microscale flow in microfluidics can reduce the reaction time and save reagents, which is desired in reactions and analyses [9,10]. On the other side of the coin, limited channel space and high-viscosity fluids make it challenging to achieve full mixing inside the microchannel [11], hindering the development of microfluidics in various applications. Many efforts have been made for mixing enhancement in microfluidics by delicately designing channel geometry [12,13,14,15,16,17,18,19,20,21], namely passive mixing, to escalate vortex generation and liquid internal perturbations. However, the increased structure complexity would induce new operational issues and be ineffective for liquids with considerably high viscosity. Therefore, active mixing, which applies external physical fields for effective mixing, has received increased attention [22,23]. These techniques apply electric, acoustic, thermal, or magnetic fields to enhance the mixing efficiency. Electric field-driven micromixers utilize the forces exerted on charged particles to improve mixing efficiency [24]; acoustic field-driven micromixers use vibrations induced by sound waves [25]; thermal field-driven mixers create convective flows for mixing enhancement [26]; and magnetic ones employ magnetic rotors driven by an external magnetic field [27,28,29,30]. While these methods result in mixing enhancement, electric field-driven micromixers risk high-voltage hazards [31], acoustic designs are costly and bulky [32], and thermal methods influence the activity of biological samples/regents [33]. Magnetic field-driven micromixers possess biocompatibility, multifunctionality, and recyclability, making them ideal for sensitive biological samples [34]. Initially, magnetic particles were employed in the micromixer, constrained by weak magnetic control, which could not sufficiently overcome the fluid’s viscous resistance [30]. To overcome these limitations, bar-shaped magnetic rotors were developed [27], and the impact of the rotor structure on mixing efficiency has drawn extensive attention.

In our prior experimental work [34], we fabricated structural magnetic nanobranches by coupling aligned Fe_3_O_4_ nanoparticles with dopamine. To investigate the influence of the rotor structure on microfluidic mixing efficiency, magnetic nanobranches and nanochains were introduced into the mixing of artificial saliva and reagent, actuated by a rotating magnetic field (rotation rates 100–600 rpm). The experimental results demonstrated that nanobranches significantly outperformed nanochains in improving mixing efficiency inside microfluidic chips. However, the experimental results still lack sufficient flow field investigation to illuminate the mechanisms underlying mixing enhancement. Furthermore, constrained by the fabrication of micro-rotors, the effects of rotor size, shape, and multi-rotor arrangements on mixing remain unexplored numerically.

Numerical simulations of micromixers mainly focus on the structural design and parameter optimizations of passive micromixers [35,36,37,38] and the promotion of mixing through physical fields like electric [39,40] and acoustic fields [41]. However, numerical studies on the promotion of mixing by the structure of the rotor and the arrangement of multiple rotors are scarce.

Here, we present a numerical study investigating the influence of rotor size, rotor shape, rotor arrangement, and rotation rate on the mixing efficiency in a microfluidic chip. The size of the rotor has a significant effect on the enhancement of mixing. The three micro-rotors with different shapes (bar-shaped, Y-shaped, and cross-shaped rotors) are applied and rotated at the contact zone of two liquids. The cross-shaped rotor shows the highest mixing efficiency among the three rotors. We further vary the arrangement of multiple rotors, showing the superior mixing performance of the cross-arrangement compared to the contact line arrangement or line arrangement of the rotors. The increase in the rotation rate (50–600 rpm) for four-rotor mixing improves the mixing efficiency, although a diminishing marginal effect is observed. This study can function as a guide for rotor design and mixing operation, offering great benefits for applications such as chemical synthesis and biological detection.

## 2. Computational Setup

### 2.1. Governing Equations

The species transport model is commonly used in the mixing of miscible fluids when considering mass diffusion [42,43,44]. The continuity equation and the momentum equation are expressed as follows [45]:

(1) $$\nabla \cdot \vec{V}=0$$

(2) $$\rho \frac{\partial \vec{V}}{\partial t}+\rho \vec{V}\cdot \nabla \vec{V}=-\nabla P+\mu \nabla^{2}\vec{V}+S$$

where $\vec{V}$, ρ, P, and μ correspond to the velocity vector, the density, the static pressure, and the kinematic viscosity, respectively. The source term *S* appearing in Equation (2) represents the effects of the rotor’s rotation. The species transport equation is formulated as

(3) $$\frac{\partial C}{\partial t}+\vec{V}\cdot \nabla C=D\nabla^{2}C$$

where C is the local concentration and D denotes the mass diffusivity.

### 2.2. Mixing Index

To evaluate the mixing efficiency, we apply the mixing efficiency index to characterize the mixing performance [43,46]. The mixing index *φ* based on the normalized standard deviation of mass fraction is calculated as follows:

(4) $$\varphi =\left(1-\frac{\sigma }{\sigma_{0}}\right)\times 100\%$$

where σ is the standard deviation of the mixing mass fraction in the computational domain:

(5) $$\sigma^{2}=\frac{1}{N}{\sum_{i=1}^{N}\left(C_{i}-\bar{C}\right)}^{2}$$

where N represents the total number of cells in the model mesh and $\bar{C}$ denotes the average mass fraction. The maximum standard deviation $\sigma_{0}$ is calculated by

(6) $$\sigma_{0}^{2}=\bar{C}(1-\bar{C})$$

The mixing index φ=1 (σ=0) indicates that two fluids are completely mixed, and φ=1 ($\sigma =\sigma_{0}$) indicates the two fluids are completely unmixed. When the φ value is high, it signifies a more consistent concentration across the sample, suggesting optimal mixing performance.

### 2.3. Physical Properties

Liquid A has a density of 1006 kg/m^3^ and a dynamic viscosity of 20 mPa·s, similar to saliva, a typical human body fluid; liquid B has a density of 1000 kg/m^3^ and a dynamic viscosity of 1 mPa·s, similar to the reagent. The mass diffusion coefficient *D* is assumed as 1 × 10^−12^ m^2^/s [47]. Within the scope of our investigation, the flows in the microchannels are characterized by a low Reynolds number (Re=ρ¯v¯Lμ¯ ~ 10^−3^, where the mean dynamic viscosity is $\bar{\mu }=10.5\;\mathrm{mPa}\cdot s$, mean fluid density $\bar{\rho }=1003\;{\mathrm{kg}/m}^{3}$, fluid velocity v=ω×r=261 μm/s, rotation of the rotor ω=100 rpm, the radius r=25 μm, and characteristic dimension L=50 μm) and a high Schmidt number ($\mathrm{Sc}=\frac{\bar{\mu }}{\bar{\rho }D}$ ~ 10^7^). Furthermore, the Peclet number is $10^{4}\gg 1$ (Pe=Re×Sc), which indicates an extremely strong advection-dominated mixing regime, where fluid motion is driven by the rotor rotation.

### 2.4. Numerical Schemes

The finite-volume method is used to discretize the mentioned equations in ANSYS Fluent. The thickness of the micromixer in our case is significantly smaller and negligible relative to its other dimensions. Miniaturization also renders the impact of gravity insignificant, and the experimentally observed mixing phenomena can be researched using a 2D model in the micromixer with the rotor [48,49]. As a result, we employed a 2D model [50,51,52,53,54,55] of a mixing chamber with rotors in our study.

In this study, we employ the species transport model and the standard *k*-epsilon model to simulate the mixing. A no-slip boundary condition is imposed on all walls, dictating that the fluid velocity is zero at the stationary wall boundary—all three velocity components (streamwise, spanwise, and normal) are strictly zero. A transient-state, double-precision implicit solver is adopted, ensuring the solution’s accuracy and stability for time-dependent flow behaviours. The rotation of the rotors is achieved by dynamic mesh, and user-defined functions (UDFs) were integrated to keep a stable rotational speed. The COUPLED algorithm scheme, which uses a combination of continuity and momentum equations to derive an equation for pressure, was applied. All spatial discrete methods used were set as second-order upwind. The converged solution is assumed when the scaled residuals of all variables are smaller than 10^−3^. In this study, with no specific mention, the time step Δt is set to 0.01 s, and the calculation is carried out for 6000 time steps.

### 2.5. Grid Independence Study

**Figure 1:**
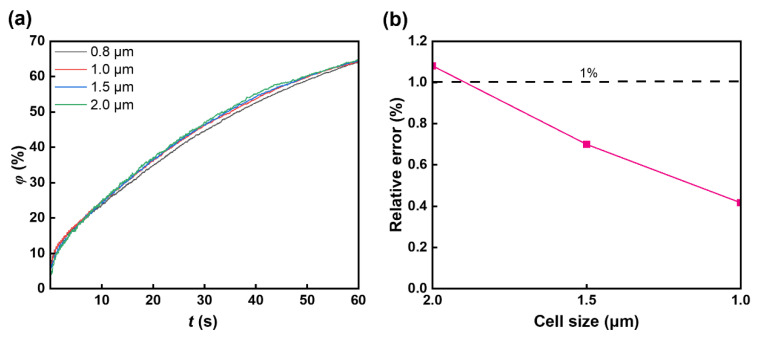
Grid independence study. (**a**) The mixing index with various cell sizes; four grid meshes with triangular cells were used: 6204 cells, 11,252 cells, 23,340 cells, and 35,126 cells. The chosen grid is that of 23,340 cells. (**b**) The relationship between cell size and relative error.

The 2D model is meshed using ANSYS Meshing. We perform the grid independence study first. The grid sizes are listed in Table 1. The relative error (RE) influenced by grid size can be described as

(7) $$\mathrm{RE}=\frac{\left|\sigma_{i}-\sigma_{4}\right|}{\sigma_{4}}\times 100\%$$

where $\sigma_{i}$ is the standard deviation in the *i*th case. Figure 1a shows the relationship between the mixing time and standard deviation with various grid sizes. When the grid size is 1 μm, the relative error is below 0.5% compared to a grid size of 0.8 μm, showing that the grid size has a negligible influence on the simulation results (Figure 1b, Table 1). Therefore, the 1 μm grid size is applied.

## 3. Results and Discussions

### 3.1. Single Rotor

**Figure 2:**
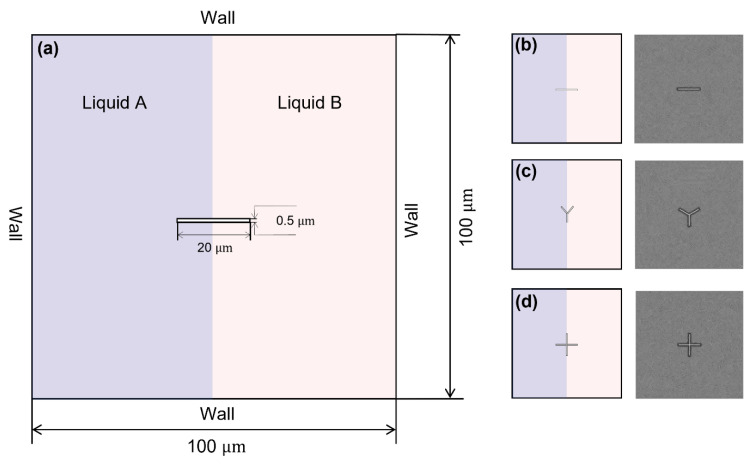
Schematic images of the mixing chamber and the rotors. (**a**) Schematic diagram of the micro-chamber; the size of the square chamber is 100 μm × 100 μm. The left part of the chamber is liquid A, and the right part is liquid B. (**b**) Bar-shaped rotor (**left**), grid of bar-shaped rotor (**right**). (**c**) Y-shaped rotor (**left**), grid of Y-shaped rotor (**right**). (**d**) Cross-shaped rotor (**left**), grid of cross-shaped rotor (**right**). The mesh around the rotor has been encrypted with a three-tiered grid refinement strategy to capture the detailed mixing dynamics.

**Figure 3:**
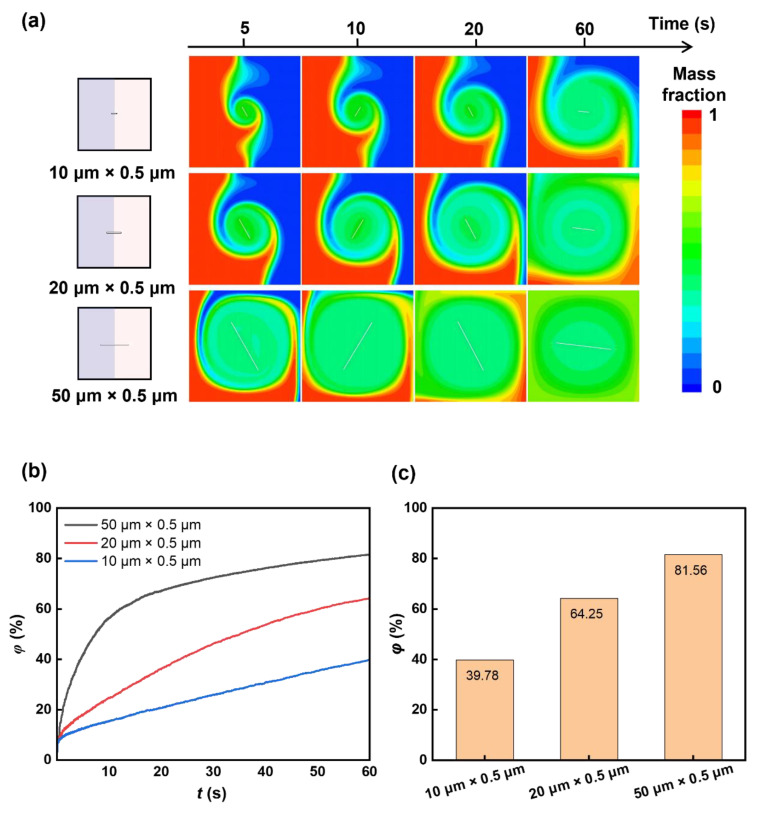
Rotor length-dependent mixing. (**a**) Sequential mass fraction distributions of rotors with different lengths during mixing. The length of the rotors is set as 10 μm, 20 μm, and 50 μm. (**b**) Temporal mixing index with rotors of different lengths. (**c**) The mixing indexes with rotors of different lengths at 60 s.

**Figure 4:**
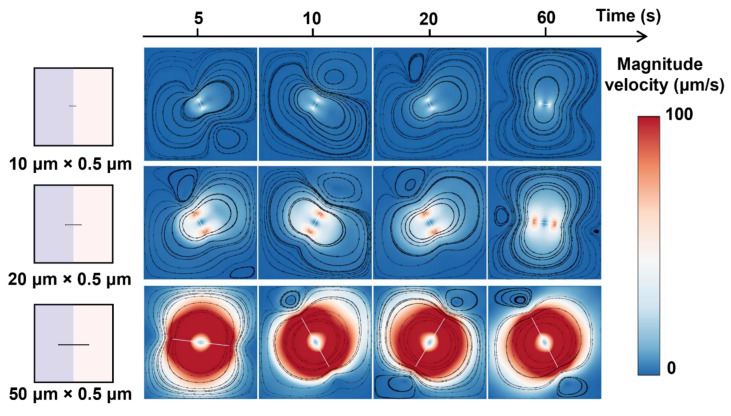
Rotor length-dependent velocity distributions during mixing. The figures overlay the velocity magnitude and streamlines within the flow field.

As shown in Figure 2a, the left part of the microfluidic chamber is liquid A, and the right part is liquid B. The rotor is at the centre of the square chamber (size 100 × 100 μm). We first investigate the mixing performance of rotors with three different lengths. Three rotors were set, with lengths of 50 μm, 20 μm, and 10 μm and the same width of 0.5 μm. The rotors rotate inside the chamber with a rotation rate of 100 rpm. The sequence of images in Figure 3a illustrates the distribution of the mass fraction of liquid A during the process of mixing. The rotor with a size of 50 μm × 0.5 μm has a more pronounced effect on the mixing of the two liquids than the rotor with a size of 20 μm × 0.5 μm and the rotor with a size of 10 μm × 0.5 μm. This enhanced mixing effect is further supported by the time-dependent mixing index, as depicted in Figure 3b,c. With the 50 μm × 0.5 μm rotor, the mixing index reaches 81.56% within 60 s, which is approximately 42% greater than that of the 10 μm × 0.5 μm rotor, highlighting the impact of rotor size on the mixing efficiency. Additionally, the velocity field images captured during rotation, as shown in Figure 4, reveal a higher flow velocity surrounding the rotor (50 μm × 0.5 μm), contributing to a higher mixing efficiency.

**Figure 5:**
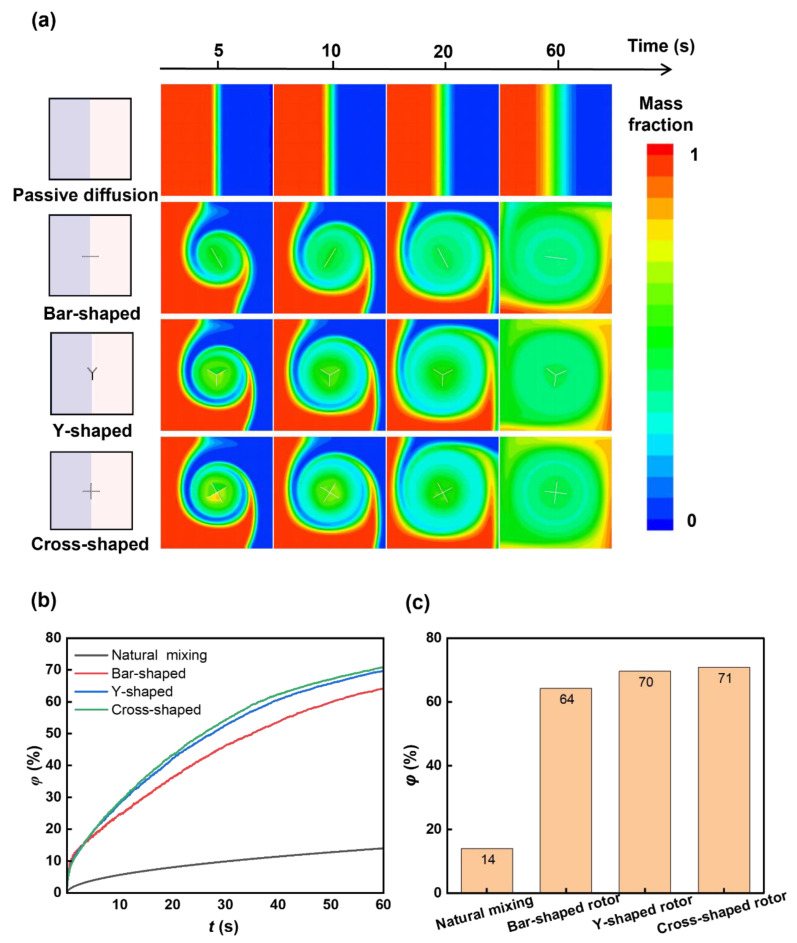
Rotor shape-dependent mixing. (**a**) Sequential mass fraction distributions of passive diffusion by bar-shaped, Y-shaped, and cross-shaped rotors during mixing. (**b**) Temporal mixing index of mixing with different rotors. (**c**) Mixing indexes of mixing with different rotors at 60 s. The mixing index with the cross-shaped rotor is higher than the bar-shaped rotor, which is in agreement with the mass fraction distributions.

**Figure 6:**
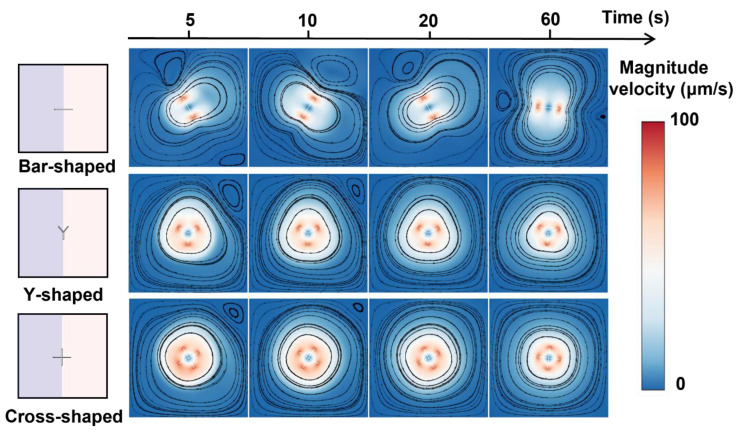
Rotor shape-dependent velocity distributions during mixing.

We next examine the mixing performance of a single rotor with different shapes. We studied the mixing performance of three kinds of rotors: bar-shaped, Y-shaped, and cross-shaped rotors with similar sizes (~20 μm × 0.5 μm, Figure 2b–d), and investigated the natural diffusion case without the rotor as a control. The sequential images in Figure 5a show the mass fraction distributions during mixing. The cross-shaped rotor shows greater influence on the mixing of two liquids compared to a bar-shaped and Y-shaped rotor, which can be further evidenced by the time-dependent mixing index (Figure 5b,c). The mixing index with the cross-shaped rotor can achieve 71% within 60 s, about 7% higher than the bar-shaped rotor and 57% higher than the natural diffusion case without the rotor, showing the influence of the rotor structure on mixing and the superior mixing enhancement of the cross-shaped rotor. The velocity field images during the rotations (Figure 6) show the higher flow velocity due to the cross-shaped rotor, resulting in higher mixing efficiency. The streamlined images (Figure 6) show a larger mixing extent of fluid around the cross-shaped rotor, enhancing the mixing efficiency. The bigger separating vortex would be generated with the bar-shaped rotor, causing lower mixing efficiency compared to the Y-shaped and cross-shaped rotors.

### 3.2. Multiple Cross-Shaped Rotors

**Figure 7:**
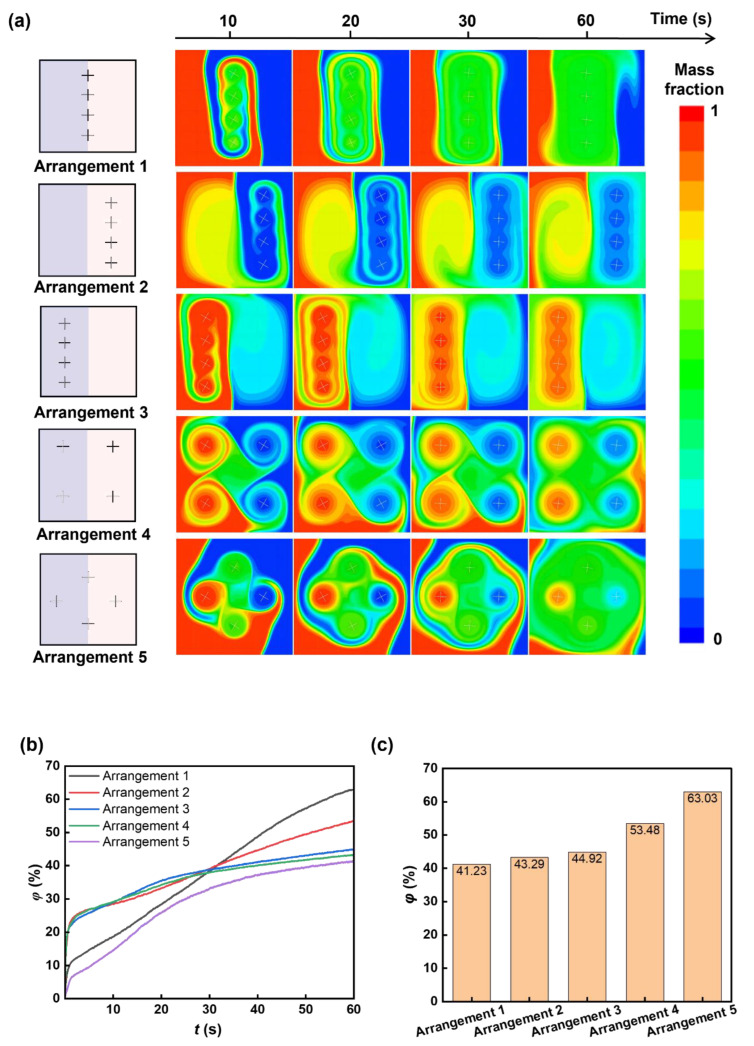
Rotor arrangement-dependent mixing. Five arrangements have been studied: rotors are all at the contact line between two liquids (Arrangement 1), rotors are all in low-viscosity liquid (liquid B, Arrangement 2), rotors are all in high-viscosity liquid (liquid A, Arrangement 3), two rotors are in low-viscosity liquid and two rotors are in high-viscosity liquid (Arrangement 4), and rotors are cross-distributed (Arrangement 5). (**a**) Sequential mass fraction distributions with different arrangements during mixing. (**b**) Temporal mixing index during mixing with different arrangements. (**c**) Mixing indexes of mixing with different rotor arrangements at 60 s.

We further study the mixing performance of multiple cross-shaped rotors. The four-time expanded square chamber (size 200 μm × 200 μm) is chosen as the mixing space, and four cross-shaped rotors (size 20 μm × 0.5 μm) with different arrangements in the chamber are tested for mixing enhancement. We use five arrangements, Arrangements 1–5 (Figure 7a), to study the influence of rotor arrangement on the mixing enhancement and determine the optimized rotor distribution. We tested all five rotor arrangements: rotors are all at the contact line between two liquids (Arrangement 1), all in low-viscosity liquid (liquid B, Arrangement 2), all in high-viscosity liquid (liquid A, Arrangement 3), two in low-viscosity liquid and two in high-viscosity liquid (Arrangement 4), and cross-distributed arrangement (Arrangement 5). As shown in Figure 7, the arrangement of rotors vastly influences the mixing performance, even though the size, structure, and number of the rotors are the same for all cases. When all rotors are at the contact line between two liquids (Arrangement 1), the lowest mixing efficiency among the five arrangements is observed, achieving a mixing index lower than 42% after a 60 s rotation (Figure 7c). When all rotors are in low-viscosity liquid, the mixing index is about 43%. When all rotors are in high-viscosity liquid, the mixing index is about 44%, which is still relatively low. When two rotors are in low-viscosity liquid and two rotors are in high-viscosity liquid, the mixing index is about 53%, and when the rotors are in the cross-distributed arrangement, the best mixing efficiency, about 63%, is observed. Distinctly, Arrangement 1 and Arrangement 5 showed lower mixing performance in the beginning because the distribution of rotors in the contact line of two miscible fluids hindered the diffusion.

**Figure 8:**
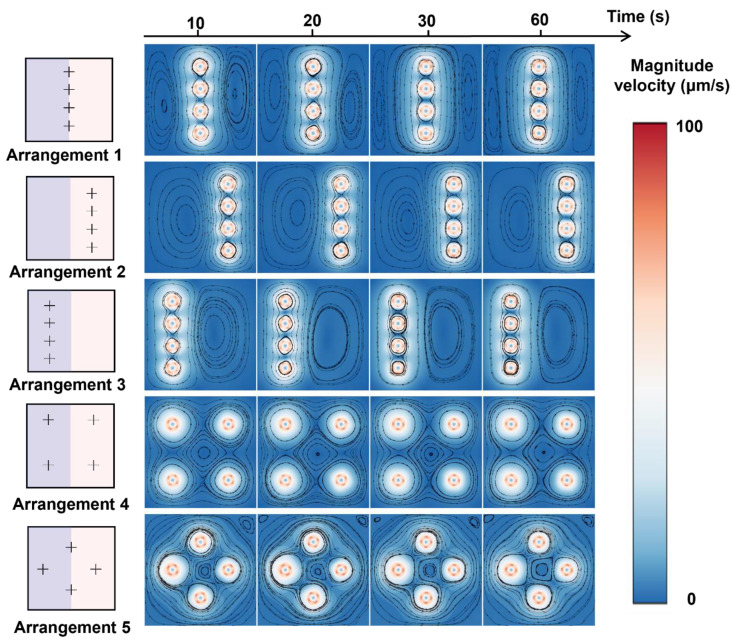
Rotor arrangement-dependent velocity distributions during mixing.

Remarkably, the cross-distributed arrangement (Arrangement 5) shows the highest mixing efficiency among the five cases, with a ~10% increase compared to the mixing efficiency of Arrangement 4. This special arrangement could prompt convection between liquid A and liquid B. The mass fraction of liquid A in Figure 7a shows that, in the initial mixing stage via Arrangement 1 and Arrangement 5, the distribution of rotors in the contact line of two miscible fluids hindered the diffusion, which contributed to a lower mixing efficiency. However, during the mixing process, the rotor distributed in the two fluids further transported the liquids to the other side, increasing the liquid contact area [56] and greatly improving the mixing efficiency. As shown in Figure 8, for the arrangements where the rotors are only distributed in a single fluid or only at the contact line of two fluids, the generated vortex area for mixing is small, and the mixing area is not greatly increased, which ultimately leads to poor mixing efficiency. When the rotor is distributed in four corners, the mixing process lacks convective transport of the two fluids, causing relatively low mixing efficiency.

**Figure 9:**
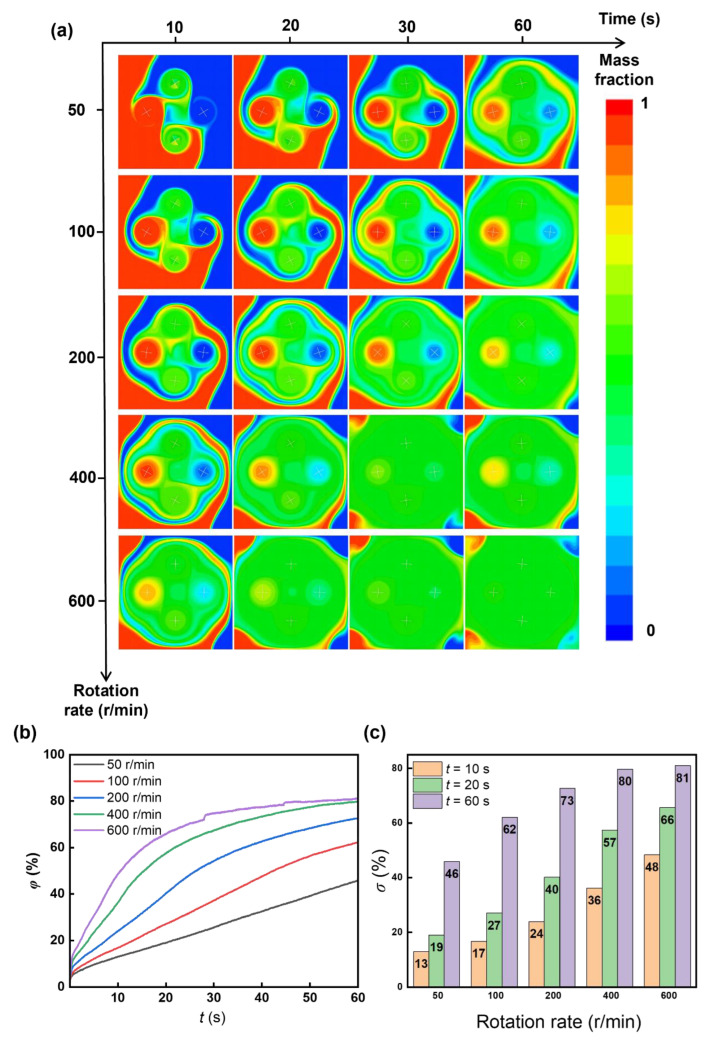
Rotation rate-dependent mixing. During the numerical solution in the rotation, the time step (Δ*t*) is modified to 0.005 s to ensure the convergence of the computational results. (**a**) Sequential mass fraction distributions for rotation rate 50–600 rpm during mixing. (**b**) Temporal mixing index during mixing with different rotation rates. (**c**) Mixing indexes with various rotation rates.

**Figure 10:**
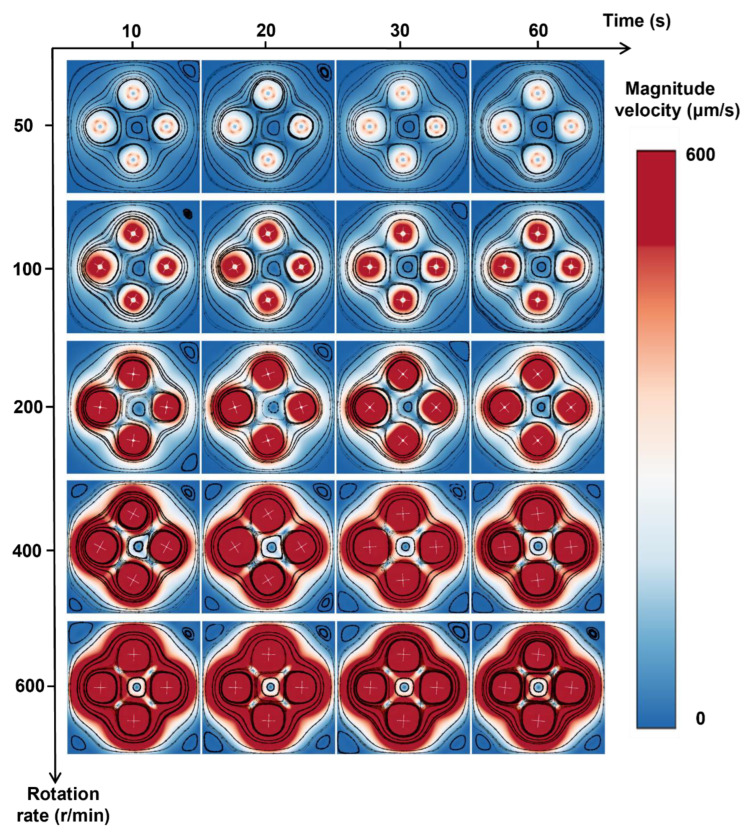
Rotation rate-dependent velocity distribution during mixing.

The rotation rate also influences the mixing efficiency. We varied the rotation rate of the cross-shaped rotor from 50 to 600 rpm and found that a higher rotation rate can effectively enhance the mixing index (Figure 9). However, an increase in the rotation rate would not improve the mixing index proportionally (Figure 9b,c and Figure 10), which means there is a diminishing marginal effect—mixing enhancement increases at a decreasing rate as the rotation rate increases. Other factors, like stability and energy input, should be taken into consideration to comprehensively determine the optimum rotation rate and experimental demand.

## 4. Concluding Remarks

The mixing performance of active mixing inside a microfluidic chip was numerically investigated. When mixing with a single rotor, the rotor structure and size affected the mixing efficiency, and the cross-shaped rotor showed higher mixing performance compared to the bar-shaped and Y-shaped rotors. When mixing with multiple rotors, we further investigated the arrangement of four rotors, finding that the cross-distributed arrangement possessed the best mixing performance among the five typical arrangements. Increasing the rotation rate improved the mixing performance, but the rate of improvement decreased as the rotation speed increased. This study serves as a valuable guide for active mixing inside microfluidics chips and can be applied to applications such as biological detection or chemical synthesis that require effective mixing.

## Figures and Tables

**Table 1:** Grids with different grid sizes.

Number	Grid Size (μm)	Number of Grids	Mixing Index (%)	Relative Error (%)
1	2.0	6204	64.67	1.08
2	1.5	11,252	64.43	0.70
3	1	23,340	64.25	0.42
4	0.8	35,126	63.98	0

## Data Availability

The original contributions presented in this study are included in the article. Further inquiries can be directed to the corresponding author.
